# Spine and Rheumatic Diseases

**DOI:** 10.1155/2015/756205

**Published:** 2015-07-28

**Authors:** James Cheng-Chung Wei, Yi Liu, Hsi-Kai Tsou, Irene Eva van der Horst-Bruinsma

**Affiliations:** ^1^Division of Allergy, Immunology and Rheumatology, Chung Shan Medical University Hospital, Taichung, Taiwan; ^2^Institute of Medicine, Chung Shan Medical University, Taichung, Taiwan; ^3^Institute of Integrative Medicine, China Medical University, Taichung, Taiwan; ^4^Department of Rheumatology and Immunology, West China Hospital, Sichuan University, Chengdo, China; ^5^Functional Neurosurgery Division, Neurological Institute, Taichung Veterans General Hospital, Taichung, Taiwan; ^6^Department of Rheumatology, EULAR Centre of Excellence, VU University Medical Centre, Room 3A-64, P.O. Box 7057, 1007 MB Amsterdam, Netherlands

Rheumatic diseases include a wide spectrum of diseases involving musculoskeletal and immune system. Spinal disorders, whether mechanical or inflammatory, are main manifestations in daily clinical practice of rheumatologists, neurologists, orthopaedics, and general physicians.

Spondyloarthritis (SpA), typically ankylosing spondylitis (AS), is one of the major rheumatic spinal disorders. The pathogenesis of SpA involved many interaction domains including genes, immunity, mechanical stress, infection, and other environmental factors [1]. Genetic backgrounds, such as HLA-B27, B60, ERAP-1, and IR23R, play major roles in the initiation and pathogenesis of SpA. These multifactorial gene-environmental interaction mechanisms lead to variable clinical phenotypes of this complex diseases spectrum, mainly arthritis in spine, sacroiliac joints, and peripheral joints, but also extra-articular manifestations, such as uveitis, psoriasis, and bowel inflammation (Figure 1).

The Assessment in Spondyloarthritis International Society (ASAS), an international task force, had generated new ASAS criteria for axial and peripheral SpA by predominant pattern of clinical manifestations in 2009 [2] and 2011 [3]. These classification criteria aimed to standardize definition of this diseases spectrum for clinical researches and emphasized early detection of SpA by usage of magnetic resonance imaging (MRI) in sacroiliac joints and human leukocyte antigen- (HLA-) B27 in diagnosis of SpA. A new disease term, nonradiographic axial SpA (nr-ax SpA), was thus created to describe patients who fulfilled the 2009 ASAS criteria for axial SpA but not the 1984 modified New York criteria for AS [4]. However, there are still many debates about the scope of this new term, nr-ax SpA, including the definition, disease natural course, clinical diagnosis, and management strategies.

Treatment goals for SpA are maintenance of physical function, disease activity, and prevention of radiographic progression to ankylosis. Current effective therapies to control SpA disease activity are available, including exercise and physical therapy [5], nonsteroidal anti-inflammatory drugs (NSAIDs), disease modifying antirheumatic drugs (DMARDs) such as sulfasalazine, and biological agents, mainly TNF blockers. Potential experimental therapies are promising, including IL17 blockers and IL23 blockers [6].

In this special issue, 16 articles were accepted, while the other 21 were rejected. The scope of this special issue covered clinical and basic studies on spinal or rheumatic diseases. A nationwide epidemiological study of hyperuricemia in Taiwan was published by C.-Y. Wei et al. Genetic studies including HLA-B27 subtype, MUTYH gene polymorphisms, SIRT1, and microRNAs were investigated by Y. Mou et al., Y.-J. Kung et al., S. Zhou et al., and Q. Lv et al. Biomarkers studies including erythrocyte C4d-to-complement receptor 1, soluble CD30, and vitamin D and pyridinoline cross-linked carboxyterminal telopeptide of type I collagen (ICTP) were done by C.-H. Chen et al., R. Gao et al., and P. Zhang et al. Clinically, anthropometric measurement of Schober's test was examined by Y.-R. Yen et al. Many articles investigating therapies, including infliximab, a novel antirheumatic drug T-614, surgical interventions, home exercise, and whole-body cryotherapy with kinesiotherapy, were addressed by Z. Lin et al., Y. Wei et al., L.-F. Hsieh et al., and A. Stanek et al. We hope this special issue covered many important aspects in inflammatory joint diseases, which will surely provide us with a better understanding about the pathogenesis, diagnosis, and treatment of spine and rheumatic diseases.

*James Cheng-Chung Wei* *Yi Liu* *Hsi-Kai Tsou* *Irene Era van der Horst-Bruinsma*

## Figures and Tables

**Figure 1:**
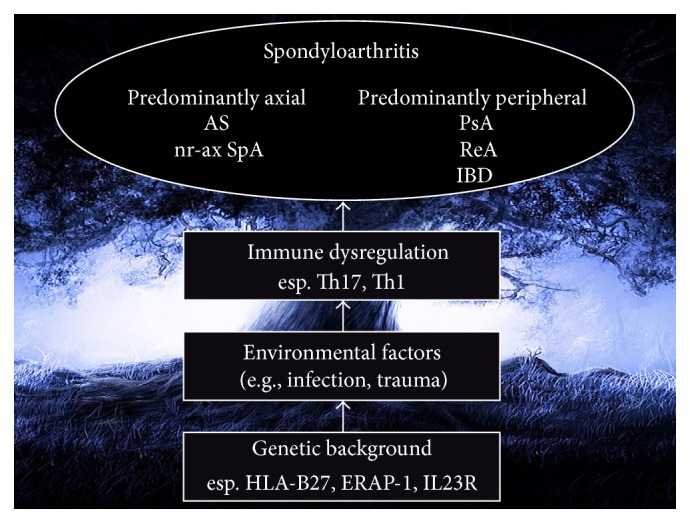
Development and pathogenesis of spondyloarthritis (SpA). Patients of SpA need a genetic background, such as HLA-B27, B60, ERAP-1, and IL23R. After being triggered by environmental factors, such as infection or mechanical trauma, the immune dysregulation and inflammation occurred in enthesis through Th17 and Th1 immune reaction. With different genetic backgrounds and environmental factors, patients may have different phenotype, such as ankylosing spondylitis, psoriatic arthritis, or inflammatory bowel diseases. AS: ankylosing spondylitis; nr-ax SpA: nonradiographic axial spondyloarthritis; PsA: psoriatic arthritis; ReA: reactive arthritis; IBD: inflammatory bowel diseases.
